# Multiscale Friction in Lubricant-Surface Systems for High-Performance Transmissions Under Mild Wear

**DOI:** 10.1007/s11249-018-1032-z

**Published:** 2018-05-24

**Authors:** E. Humphrey, N. Morris, M. Leighton, R. Rahmani, H. Rahnejat

**Affiliations:** Wolfson School of Mechanical, Electrical and Manufacturing Engineering, Loughborough University, Loughborough, UK

**Keywords:** Gear, Tribo, Film, Additive, ZDDP, AFM, Atomic force microscopy, Wear, Friction

## Abstract

The lubricant-surface system is complex in nature and can significantly affect the frictional performance of high-performance transmission systems. The complexity stems from the coupled mechanical and chemical phenomena that occur at the interfacial tooth conjunctions. A combined analytical and precision experimental approach is presented to analyse the salient parameters of the lubricant-surface system. A multiscale procedure comprising topographical measurement, pin-on-disc tribometry, atomic force microscopy in lateral force mode, X-ray photo-electron spectroscopy and continuum contact mechanics analysis under mixed non-Newtonian thermo-elastohydrodynamics is used to describe the formation of a tribo-film, as well as wear and frictional characteristics of the lubricant-surface system. The contribution of chemisorbed and physisorbed bonded tribo-film on the boundary coefficient of friction is ascertained at different physical scales. Therefore, the paper presents a novel multiscale analysis, promoting improved understanding of the complex interactions between mechanisms of friction, wear and surface chemistry.

## Introduction

Improved energy efficiency of transmission systems is a key objective in high-performance vehicles. The losses attributed to friction include those due to meshing of gear pairs, their bearing supports, windage and drag within the air-oil mist environment of transmission casing, as well as pumping losses. These losses directly promote increased fuel use and harmful emissions, for which increasingly stringent directives are legislated. Therefore, the drive for increased efficiency involves all the vehicular powertrain components. For commercial road vehicles, the suggested efficiency improvements are between 3 and 5% through mitigation of friction and 4–5% for dual clutch and transmissions [1]. Research and development in technologies, purporting to improve system efficiency, commonly occur through the ‘track-to-road’ concept [2]. High-performance transmissions are routinely subjected to extreme contact pressures, temperatures and kinematics of contacting teeth pairs [3]. Owing to these prevailing extreme contact conditions, it is critical to fundamentally understand the effect of governing operating parameters, before suitable remedial actions can be proposed.

Major advances in transmission efficiency have already taken place through the evolving understanding of mixed elastohydrodynamic regime of lubrication at high loads, often yielding thin films subjected to non-Newtonian shear [4–10]. A recent trend for improving high-performance transmissions is the development of dry sump concept, which reduces lubricant drag and windage losses, as well as reducing direct emissions from the transmission housing. Lowering of lubricant viscosity also helps in reducing pumping losses by scavenging small volumes of lubricant from the sump and recirculating via impinging directed oil jets. These actions can lead to lubrication problems. Therefore, high pressure, high shear characteristics of formulated lubricants [11] have been determined and used in elastohydrodynamic analysis. The conditions often prevalent in practice such as conjunctional inlet shear heating and starvation can then be considered in a realistic manner [12]. With reduced lubricant viscosity and ultra-thin films, subject to high pressure and shear, the role of surface-active lubricant additives has become crucial. Bench top tribometers, operating under mixed and boundary regimes of lubrication have been used to create conditions which are conducive to activation of additives and formation of desired tribo-films [13, 14]. Contact mechanics and frictional characterisation of tribo-films are, therefore, essential in development of predictive methods.

The advent of nanoscale mechanical testing using Atomic Force Microscopy (AFM) has provided an impetus in the fundamental understanding of tribo-film formation and mechanical behaviour [15–18]. Pidduck and Smith [18] showed that Lateral Force Mode (LFM) with AFM can be used to investigate tribo-films formed through tribometric activation. Carpick et al [19, 20] showed that measurements taken in a vacuum for nanoscale frictional properties, using LFM, are functions of contact area, adhesion, and surface shear strength. Umer et al [21] used a similar approach to benchmark automotive cylinder coatings using these nanoscale mechanical properties, including the shear strength and modulus of elasticity.

Nanoscale analysis has been used to determine the mechanical properties of thermal and thermo-mechanically generated tribo-films [22–24]. Bec et al [22] used a surface force apparatus to investigate the anti-wear properties of Zinc Dialkyldithiophospates (ZDDP) films, such as the rate of film formation and its shear strength. Variations in nanoscale modulus of elasticity of ZDDP films were shown by Graham et al [23], using AFM. The mechanism and rate of tribo-film growth have been shown to be important for mechanical and thermally activated composition of ZDDP tribo-films. Kim et al [25] used a nano-indenter to investigate tribo-film hardness and elasticity of an ashless extreme-pressure additive generated through tribological tests. Topolovec-Miklozic et al [26, 27] used a mini-traction machine (MTM) to investigate ZDDP tribo-film formation on DLC. After the surface was lightly cleaned with propanol, surface height and lateral force images measured using an AFM were used to gain an understanding of the tribometric results. Zhang and Spikes [28] investigated the mechanisms of anti-wear tribo-film formation using topographical imaging of the surface and showed the evolution of surface topography with the growth of the tribo-film.

The current paper uses LFM to investigate the mechanics of the tribo-film formed through application of representative pin-on-disc tribometry. Measurement of frictional characteristics in conjunction with surface wear and surface chemical analysis provides a detailed understanding of the complex multiscale interactions which influence the parasitic losses within high-performance transmission systems.

## Experimental Methodology

### Overview

A procedure, including a testing protocol, is established to enable precise and repeatable measurements within an integrated experimental–numerical analysis. This includes inspection of surfaces of test specimen prior and post tribometry to determine any topographical and chemical compositional changes, as well as frictional characteristics. Topographical measurements are carried out using an optical focus variation technique with the Alicona focus variation microscopy with a nominal vertical resolution of 10 nm and horizontal resolution (along the surface directions) of 0.44 µm. X-ray photoelectron spectrometry (XPS) is used for chemical analysis of tribo-films formed on the specimen surface. LFM is used to measure frictional characteristics of these evolving tribo-films, which are subject to growth as well as wear during tribometry. The results of measurements are then combined with an analytical continuum contact mechanics model to accrue further fundamental understanding of the results of experiments, particularly the frictional performance of formed tribo-films.

### Tribometry

**Fig. 1:**
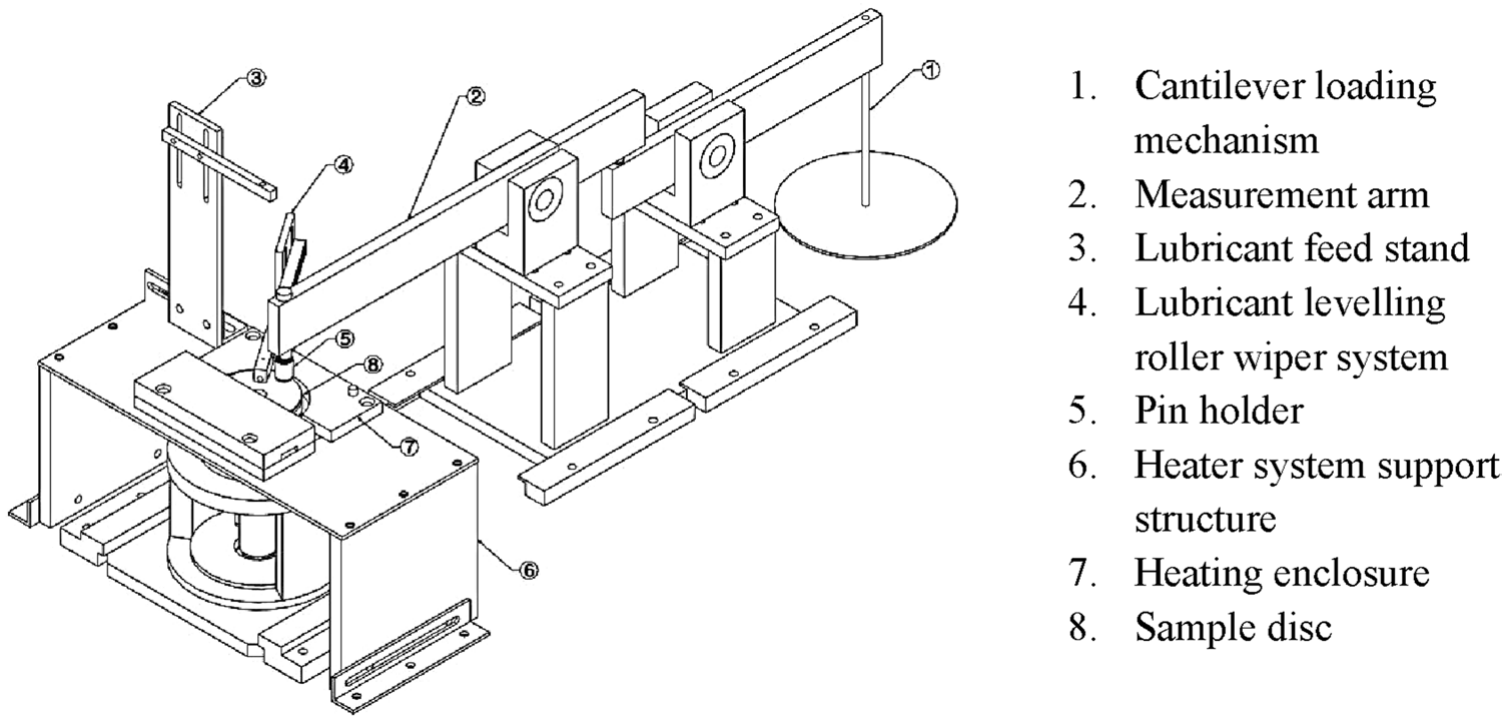
A schematic diagram of the in-house unidirectional precision pin-on-disc tribometer

A precision pin-on-disc tribometer, Fig. 1, is used to replicate some aspects of meshing teeth pair cycles of a high-performance transmission. The normal contact force applied onto the sample disc surface (5) is generated by a cantilever loading mechanism (1). The rotation of the sample disc causes relative sliding between the two surfaces (pin and disc). Generated friction is measured by a strain gauge rosette mounted upon the cantilever arm. An enclosed heating system (6 & 7) is used to raise, maintain and determine the bulk temperature of the disc samples. A K-type thermocouple is used to create a feedback loop. Lubricant is fed into the contact using a fixed feed rate syringe driver and a roller wiper system is used to maintain a layer of lubricant of constant thickness on the surface of the disc, which acts as a consistent inlet meniscus height for all tests (4). The material and basic topographical data for the pin and disc samples are listed in Table 1. To manufacture the pin, AISI52100 grade bearing steel with high chromium content and readily commercially available with similar properties to the gear steel in high performance racing transmissions is used. The disc is made from a typical gear steel (EN36C) which is very similar in mechanical properties to the pin. The discs are case carburised to a hardness of 700 HV.

**Table 1:** Material properties

Component /material	Property	Value	Unit
Pin—AISI52100 (Grade 10)	E	212	$GPa$
	Poisson’s ratio	0.28	–
	Hardness	700–900	$HV$
	RMS (Rq)	0.11	$\mu m$
	Skewness (Ssk)	− 0.0632	–
	Kurtosis (Sku)	2.66	–
Disc—EN36C (Case carburised)	E	210	$GPa$
	Poisson’s ratio	0.3	–
	Hardness	$700$	$HV$
	RMS (Rq)	$0.24$	$\mu m$
	Skewness (Ssk)	0.0402	–
	Kurtosis (Sku)	3.30	-

Sample preparation is a key stage within the procedure as the presence of any contaminants on the surface would affect the integrity and repeatability of measurements. A non-polar solvent, petroleum ether, is used to thoroughly clean all the sample surfaces.

It is essential to characterise the contact of teeth pairs in a meshing cycle of the investigated high-performance transmission. This enables the determination of necessary pin-on-disc operational conditions (contact load, contact pressure and sliding velocities) to closely replicate that of the intended system as far as possible. It should be noted that pin-on-disc experiments promote pure sliding contact conditions, whereas the involute gear teeth of the investigated transmission are subject to slide-roll meshing condition. However, tribometry is primarily used to activate the lubricant’s boundary active elements. Typical operating parameters for a simulated high-performance transmission system have been used [29]. The meshing cycle is compared with the range of operating conditions generated on the tribometer, using the Greenwood chart [30] and Deborah number [31].

The dimensionless parameters used in the Greenwood chart indicate the prevailing regime of lubrication. These are the dimensionless elastic,$~{G_e}$ , and viscous, ${G_v},$ parameters:

1 $${G_e}=\frac{{{W^*}^{{{\raise0.7ex\hbox{$8$} \!\mathord{\left/ {\vphantom {8 3}}\right.\kern-0pt}\!\lower0.7ex\hbox{$3$}}}}}}{{{U^*}^{2}}}$$

2 $${G_v}=\frac{{{G^{\text{*}}}{W^{\text{*}}}^{3}}}{{{U^{\text{*}}}^{2}}}$$

where the dimensionless speed (rolling viscosity), load, and materials’ parameters are as follows:

3 $${W^{\text{*}}}=\frac{W}{{{E^{\text{*}}}{R^2}}},~{U^{\text{*}}}=\frac{{{U_e}\eta }}{{{E^{\text{*}}}R}},~{G^{\text{*}}}={E^{\text{*}}}{\alpha _0}$$

4 $$\frac{1}{{{E^*}}}=\frac{1}{{\left( {\frac{{{E_1}}}{{1 - {\upsilon _1}^{2}}}} \right)}}+\frac{1}{{\left( {\frac{{{E_2}}}{{1 - {\upsilon _2}^{2}}}} \right)}},$$

where *W* is the applied normal contact load. In the case of meshing gear teeth, the value of *W* changes through the meshing cycle. This is obtained from an analysis of the same transmission, reported by Fatourehchi et al [3]. In the case of pin-on-disc tribometry, *W* is the applied contact load through the pin, and because of the hemispherical profile of the pin contact face, the length *L* in definition of ${W^*}$ is replaced by the pin contact face radius, *R*. ${U_e}$ is the speed of lubricant entrainment into the contact; $R$ is the equivalent radius of the meshing teeth pair along the semi-minor axis of their instantaneous contact during a meshing cycle (for the case of gears) and the pin radius in the case of tribometry; $\eta$ is the dynamic viscosity of the lubricant used. ${E^*}$ is the effective (reduced) Young’s modulus of elasticity of the contacting pair, where ${E_{1,2}}$ and ${\upsilon _{1,2}}$ are their moduli of elasticity and Poisson’s ratios, respectively.

The dimensionless elastic and viscous parameters are calculated to be in the range: ${G_e}={10^5} - ~{10^{11}}$ and ${G_v}={10^7} - {10^{13}}$ for the high-performance transmission meshing cycle and ${G_e}={10^8} - {10^9}$ and ${G_v}={10^9} - ~{10^{10}}$ for the pin-on-disc tribometry. The analysis shows that the contact condition of the transmission resides within Piezo-viscous Elastic (elastohydrodynamic) regime of lubrication. By using a 10-mm-radius hemispherical pin, a sufficiently small Hertzian contact footprint is formed, which allows for representative contact pressures ($1 - 3~GPa$) to be attained. Moreover, the high values of the dimensionless viscous parameter indicate high contact shear conditions and non-Newtonian lubricant behaviour.

The high values of ${G_v}$ parameter at its upper bound range indicate non-Newtonian shear of the lubricant. However, as the contact footprint semi-minor half-width with the tribometer is typically smaller than that of the meshing teeth pairs, it is necessary to ascertain the lubricant response time in both cases. The Deborah number [31] is calculated for both the actual gear teeth pair contact as well as that of pin-on-disc to ensure that both contacts are within the same regime of lubricant traction and also help describe the extent of non-Newtonian shear behaviour. The Deborah number is described as the ratio of the lubricant relaxation time, $\left( {\eta /G} \right),$ to the time of its passage through the contact in the direction of entraining motion, $\left( {{U_e}/2a} \right)$ [31], thus

5 $$D=~\frac{{\eta {U_e}}}{{2aG}}$$

where $a$ is the Hertzian contact semi-half width in the direction of sliding, and $G$ is the lubricant shear modulus. The lubricant rheological state (*η, G*) is adjusted for the prevailing contact pressure and temperature. For lubricant viscosity [32, 33],

6 $$\eta ={\eta _0}{e^{({\alpha ^*}p)}}$$

7 $${\alpha ^*}=\frac{1}{p}\left[ {\ln \left( {{\eta _0}} \right)+9.67} \right]\left\{ {{{\left( {\frac{{{\Theta _e} - 138}}{{{\Theta _0} - 138}}} \right)}^{ - {S_0}}}{{\left( {1+\frac{p}{{1.98 \times {{10}^8}}}} \right)}^z} - 1} \right\}$$

where $~{\alpha ^*}$is a function of both pressure and temperature. ${S_0}$ and $z$ are constants for the lubricant which are independent of the system pressure and viscosity. ${{{{\Theta}}}_e}$ is the effective contact temperature and ${{{{\Theta}}}_0}$ is a reference temperature at ambient atmospheric conditions.

8 $$z=\frac{{{\alpha _0}}}{{5.1 \times {{10}^{ - 9}}[\ln \left( {{\eta _0}} \right)+9.67]}},~~{S_0}=\frac{{{\beta _0}({\Theta _e} - 138)}}{{\ln \left( {{\eta _0}} \right)+9.67}}$$

${\alpha _0}$ and ${\beta _0}$ are the pressure and temperature coefficients for the lubricant at ambient conditions, respectively.

For lubricant shear modulus [34],

9 $$G=\left( {{G_0}+{\alpha _0}p} \right){e^{\left\{ {{\beta _0}\left( {\frac{1}{{{\Theta _e}}} - \frac{1}{{{\Theta _0}}}} \right)} \right\}}}.$$

Using the adjusted lubricant rheological state, (*η, G*), the Deborah number is found to be $D \gg 1$ throughout the meshing cycle of the high-performance transmission, indicating non-Newtonian traction, which confirms the above analysis using the Greenwood chart [30]. For the pin-on-disc experiments the Deborah number falls in the range: 1–4.835. Therefore, it can be concluded that the lubricants response under the conditions created through tribometry is a good replication of those in situ within the transmission gearing contacts. As with the Greenwood chart analysis, the main limiting factor with tribometry is the lubricant entrainment velocity. Table 2 lists the evaluated tribometer test conditions.

**Table 2:** Tribometer conditions

Parameter	Value	Units
Sliding speed	0.8–5	$m/s$
Bulk surface Temperature	333	$K$
Contact pressure	1.16	$GPa$

## Continuum Contact Mechanics

**Fig. 2:**
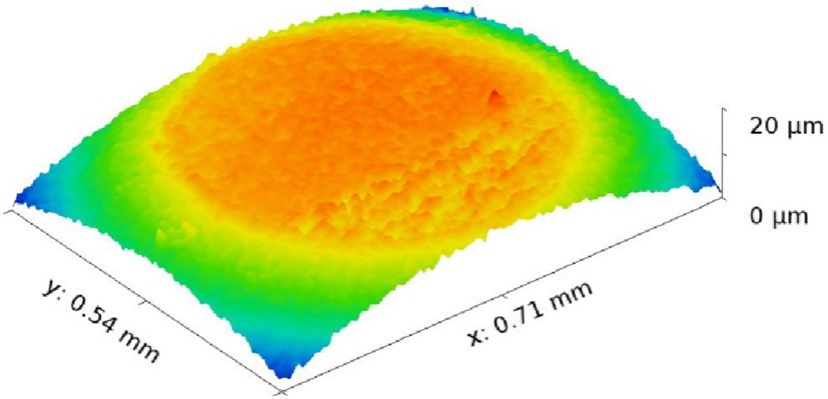
Focus variation interferometric image of the wear spot on the hemispherical profiled pin

An analytical continuum contact mechanics model is used for micro-scale point contact of the pin and the disc. This model is used for a better understanding of the experimental results. The analytical model also links the scale-dependent measured parameters of the lubricant-surface system which include contact profile, surface topography and pressure coefficient of boundary shear strength. The effect of adhesion is negligible in the current analysis, as the surface energy is significantly reduced in the presence of a fully formulated lubricant. The results for load parameter $\bar {p}=\frac{P}{{\pi wR}} \approx 8 \times {10^3}$ for the case studied here, which puts the conditions in the Hertzian region of the adhesion chart of Johnson and Greenwood [35]. Thus, the contact pressure and footprint dimensions are determined using the classical Hertzian theory [36, 37].

10 $$A=\pi {\left( {\frac{{3WR}}{{4{E^*}}}} \right)^{2/3}},$$

where $A$ is the apparent contact area. This is also measured from the wear spot made on the pin surface, using optical focus variation technique (Fig. 2). Therefore, the Hertzian contact pressure is obtained as follows:

11 $${p_0}=\frac{3}{2}\bar {p}={\left( {\frac{{6W{E^*}^{2}}}{{{\pi ^3}{R^2}}}} \right)^{\frac{1}{3}}}.$$

Hertzian theory is based upon contact of a pair of nominally smooth frictionless elastic ellipsoidal solids of revolution. However, real engineering surfaces are rough, and the interaction of their ubiquitous asperities in relative motion generates friction. To determine the boundary friction due to these interactions, it is necessary to calculate the asperity contact area and the load carried by the asperity tip contacts [38]:

12 $${A_a}={\pi ^2}{\left( {\xi \beta \sigma } \right)^2}A{F_2}\left( \lambda \right)$$

13 $${W_a}=\frac{{16\sqrt 2 }}{{15}}\pi {\left( {\xi \beta \sigma } \right)^2}\sqrt {\frac{\sigma }{\beta }} {E^*}A{F_{{\raise0.7ex\hbox{$5$} \!\mathord{\left/ {\vphantom {5 2}}\right.\kern-0pt}\!\lower0.7ex\hbox{$2$}}}}\left( \lambda \right),$$

where ${W_a}$ is the share of applied contact load carried by the asperities and$~{A_a}$ is the total asperity contact area; $\beta$ is the average asperity tip radius; $\sigma$ is the composite RMS roughness of the contacting surface, and $\xi$ is the average asperity peak density of the surface. These parameters are measured using the Alicona focus variation microscope. Equations (12) and (13) are based on the assumption of a Gaussian distribution of asperity peak heights. This is not true however for all engineering surfaces and non-Gaussian surfaces surface-specific distributions can be obtained through interferometry [39]. The topographical measurements in Table 1 show a kurtosis, ${\text{Sku}}\sim 3$ and a skewness of ${\text{Ssk}}\sim 0$, indicating Gaussian asperity height distribution and justifying the use of method expounded in [38]. In the current analysis, the surface-specific parameters such as the average asperity tip radius, RMS roughness and peak density are measured from the region of the wear scar in Fig. 2, when steady-state condition is reached after an initial period of running-in wear. The roughness parameter $\left( {\xi \beta \sigma } \right)=6.99 \times {10^{ - 3}}$ and the ratio that provides the average asperity slope [37] $\left( {\sigma /\beta } \right)=3.99 \times {10^{ - 2}}$. The statistical functions ${F_2}\left( {{{\uplambda}}} \right)$ and ${F_{5/2}}\left( {{{\uplambda}}} \right)$ are functions of the Stribeck’s oil film parameter: λ = *h*_0_/σ. These are often approximated by a polynomial function of ${{{\uplambda}}}$ [40].

### Lubricant Film Thickness

The lubricant film thickness can be obtained under the instantaneous contact conditions pertaining to the operations of tribometry using an extrapolated lubricant film equation, obtained through regression of a large number of numerical predictions. One such equation, used in the current analysis, is that of Chittenden et al [41]:

14 $${H_0}^{*}=4.31{\left( {{U_e}^{*}} \right)^{0.68}}{\left( {{W_e}^{*}} \right)^{ - 0.073}}{({G_e}^{*})^{0.49}}\left\{ {1 - {e^{\left[ { - 1.23{{\left( R \right)}^{0.67}}} \right]}}} \right\},$$

where the non-dimensional parameters are as follows:

15 $${H_0}^{*}=\frac{{{h_0}}}{R},~{U_e}^{*}=\frac{{{\eta _0}{U_e}}}{{{E^*}R}},~{W_e}^{*}=\frac{{{W_v}}}{{{E^*}{R^2}}},~{G_e}^{*}=~{E^*}{\alpha _0}$$

where $R~$is the radius of curvature of the hemispherical pin; ${\eta _0}$is the atmospheric lubricant dynamic viscosity; ${U_e}$ is the lubricant entrainment velocity; $\alpha$ is the lubricant pressure viscosity index, and ${h_0}$ is the central film thickness. Equation (13) is applied to the portion of contact footprint, which is lubricated and subject to elastohydrodynamic regime of lubrication. Thus, ${W_v}$ is the share of load carried by the lubricant film. This, in addition to the share of load carried by direct asperity pair contacts, ${W_\alpha }$, should equate the applied contact load for instantaneous quasi-static equilibrium. As ${W_v}$ is not known *a priori*, then all the non-dimensional groups in Eq. (15) are substituted into Eq. (14). The resulting equation is re-organised, yielding a relationship of the form:

16 $${W_v}=Kh_{0}^{{ - 13.7}},$$

where *K* is a function of other system parameters in the dimensionless groups of Eq. (15), which are constant. The only other unknown in the resulting equation is the film thickness, ${h_0}$. The following iterative procedure is used:

Step 1: An initial guess is made for the value of ${h_0}$ and ${W_v}$ is obtained from Eq. (16).

Step 2: For a given value of the Stribeck’s oil film parameter, λ, the share of contact load carried by the asperities is obtained from Eq. (13).

Step 3: The contact reaction is obtained as follows: $={W_v}+{W_\alpha }$. The contact reaction should equate the applied contact load (pin load), *W*, within a specified error tolerance.

Step 4: The convergence criterion is

17 $$\frac{{F - W}}{W} \leqslant \varepsilon,$$

where an error tolerance of $\varepsilon ={10^{ - 10}}$ is used in the current analysis.

Step 5: If the convergence criterion is not met, then the film thickness is adjusted as follows:

18 $$h_{0}^{n}=(1+B\vartheta )h_{0}^{{n - 1}},$$

where $n$ is an iteration counter, and $B$ is a damping factor with a typical value of 0.005 for this analysis, and $\vartheta =\frac{{F - W}}{{{\text{max}}\{ W,F\} }}$.

Then, steps 2 through 4 are repeated until the convergence criterion in Eq. (17) is satisfied.

### Viscous Friction

The generated contact friction is partly due to the interaction of asperities on the counter face surfaces (i.e. pin and disc) and partly because of viscous shear of a thin lubricant film. The analysis described above has already shown that the shear of the thin lubricant film follows non-Newtonian behaviour (i.e. Deborah number, $D>1$). Evans and Johnson [42] provided a relationship, based on combined measurement and analysis for viscous coefficient of friction of thin films subjected to non-Newtonian shear:

19 $${\mu _v}=0.87{\alpha _0}{\tau _0}+1.74\frac{{{\tau _0}}}{{\bar {p}}}\ln \left[ {\frac{{1.2}}{{{\tau _0}{h_0}}}{{\left( {\frac{{2{K_{(L)}}{\eta _0}}}{{1+9.6\xi }}} \right)}^{0.5}}} \right],$$

where ${\tau _0}$ is the lubricant characteristic Eyring shear stress, which is 4 MPa for the lubricant used in the current study; $\bar {p}$ is the average (Pascal) contact pressure; ${K_L}=~0.148~\left( {W/mK} \right)$ is the lubricant’s thermal conductivity; $,$ ${\alpha _0}=1.69 \times {10^{ - 8}}~Pa.{s^{ - 1}}~$is the lubricant pressure-viscosity index, and $\xi$ is given as follows [42]:

20 $$\xi =\frac{4}{\pi }~\frac{K}{{{h_0}/R}}{\left( {\frac{{\bar {p}}}{{{E^*}R{K_{(s)}}{\rho _{(s)}}{c_p}_{{(s)}}{U_e}}}} \right)^{1/2}},$$

where ${K_{(s)}},~{\rho _{(s)}},~{c_p}_{{(s)}}$ are the thermal conductivity, density and specific heat capacity of the bulk solid surface material. For the samples used,${K_{(s)}}=52~W/K$, ${\rho _{(s)}}=7850~kg/m$ and$~{c_p}_{{(s)}}=460~J/(kg.K)$.

Therefore, viscous friction is obtained as follows:

21 $${f_v}={\mu _v}{W_v}.$$

### Boundary Friction

Under the prevailing mixed regime of lubrication, some contribution due to boundary friction occurs as the result of counter face asperity interactions [43]:

22 $${f_b}={\tau _b}{A_a},$$

where ${\tau _b}$ is the boundary shear stress of surface/tribo-film [43]:

23 $${\tau _b}=~{\tau _0}+~\zeta \bar {p},$$

where $\zeta$ is the pressure coefficient of boundary shear strength and is measured using LFM (Sect. 4.2). ${\tau _0}~$is the Eyring shear stress of the lubricant, which is 2.5 MPa in this case. $\bar {p}$ is the mean pressure of contacting asperities:

24 $$\bar {p}=\frac{{{W_a}}}{{{A_a}}}$$

The high normal contact pressures $\bar {p} \approx 1 - 5~GPa$ found at the interface between asperities determine the characteristic boundary shear stress of the surface/tribo-film which is significantly greater than the Eyring shear stress of the lubricant at the onset of non-Newtonian behaviour $\left( {{\tau _0}\sim 2.5~MPa} \right)$. Thus, ${\tau _b} \approx ~\zeta ~\bar {p}$. The values for the pressure coefficient of boundary shear strength $(\zeta )$ are measured using AFM in lateral force mode (Sect. 4.2).

Therefore, the total generated friction becomes

25 $$f={f_v}+{f_b}.$$

### Temperature Rise Within the Contact

A proportion of input energy is dissipated through generated friction in the form of heat. This is used to readjust the contact temperature and therefore the viscosity and shear modulus of the lubricant in an iterative process. The contact flash temperature can be obtained as a mean temperature rise for the circular point contact of the hemispherical pin against the flat disc as [44, 45]

26 $${\Theta _m}=\left( {\frac{{0.31fU}}{{ka}}} \right)\left( {\sqrt {\frac{k}{{\rho {c_p}Ua}}} } \right),$$

where ${\text{f}}$ is the total friction from a previous iteration step (Eq. (25)). Equation (26) is shown to be accurate when the dimensionless speed parameter ${\beta ^*}>5$, where ${\beta ^*}$is defined as [44, 45]

27 $${\beta ^*}=\frac{{Ua{\rho _{(s)}}{c_p}_{{(s)}}}}{{2{K_{(s)}}}}.$$

The mean temperature obtained from Eq. (26) exceeds the bulk temperature of the disc surface ${{{\Theta}}}$ maintained within the heating enclosure (Fig. 1), and thus the effective contact temperature (used to adjust lubricant viscosity and shear modulus) becomes:

28 $${\Theta _e}={\Theta _m}+~\Theta.$$

This method uses both analytical equations and measured surface-specific parameters to enable accurate prediction of the complex lubricant-surface system frictional characteristics.

## Results and Discussion

### Micro-scale Topographic and Tribometric Measurements

**Fig. 3:**
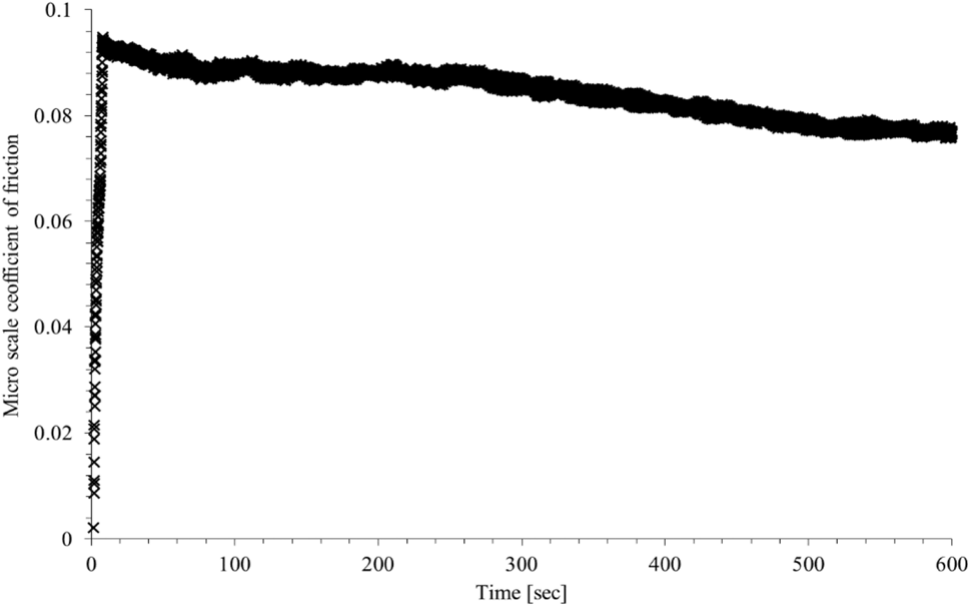
An example of friction trace at sliding speed of 0.8 m/s

Frictional performance of the EN36C disc sample with a commercial transmission lubricant (80W90 grade, with a kinematic viscosity of $~140m{m^2}/s$at 40 °C) is ascertained using a precision in-house pin-on-disc tribometer (Fig. 1). Further description of the tribometer is provided in Sect. 2.2. The average coefficient of friction is taken from the nominal steady-state portion of the measured data in Fig. 3.

**Fig. 4:**
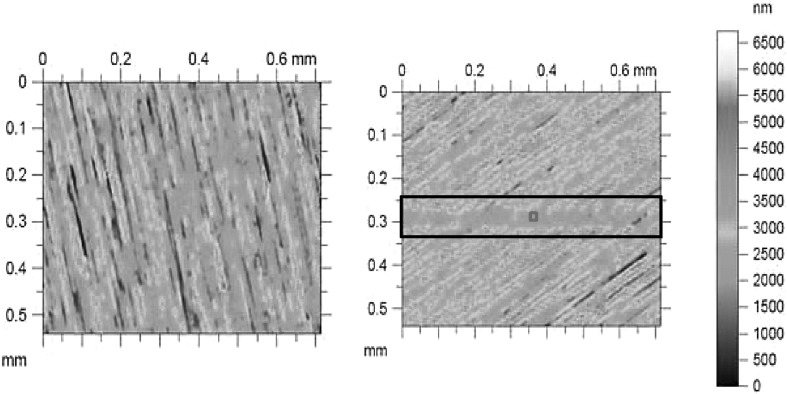
Focus variation microscope images of a disc sample pre and post tribometry: (left) virgin surface, (right) worn sample with indicated wear scar

**Fig. 5:**
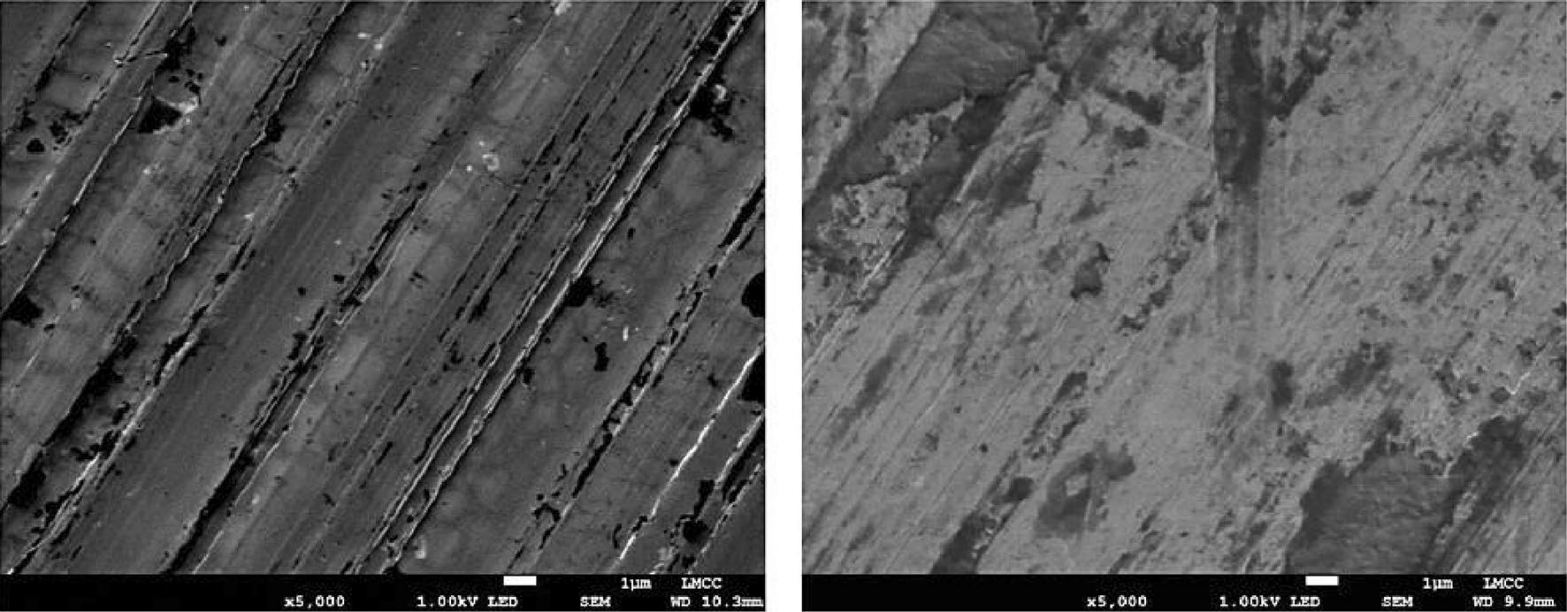
SEM images of sample surfaces pre and post tribometry: (left) virgin sample, (right) post tribometry within the wear scar region of shown on Fig. 4

Figures 4 and 5 show surface topographical images using focus variation microscopy, as well as scanning electron microscopy (SEM) of a disc sample surface prior to and post tribometry. They show the evolution of surface topography. The images show that steady-state conditions are reached after an initial period of running-in wear has elapsed. This is shown within the indicated boxed region of the post-tribometric image, identifying the wear scar on the disc surface in Figs. 4 and 5.

### Nanoscale Measurements

Post tribometry the samples are analysed for nanoscale boundary friction with LFM, following the detailed approach described by Leighton et al [17]. This is to determine the interfacial pressure coefficient of boundary shear strength between asperity contacts, ζ and the nanoscale boundary friction properties. These values are used as input data to the analytical continuum contact mechanics model described in Sect. 3. LFM enables measurement of these parameters on the disc samples before and after tribometry. Thus, the effect of any activated and retained tribo-film can be ascertained. A new approach is adopted to allow for analysis of any formed physisorbed or chemisorbed layers of an activated tribo-film. LFM is used to analyse the wear tracks on the disc sample surfaces post tribometry. The wear tracks are chosen as they represent areas of direct interactions of the contiguous contacting surfaces subject to shear, thermal and pressure activation of the lubricant additives at different contact sliding speeds. A $1~\mu {m^2}$ section of the wear scar, shown in Fig. 4 (red square—not to scale), is scanned using 1024 samples per line with 256 lines. A DNP-10 AFM probe with a triangular cantilever of spring stiffness 0.12 N m^− 1^ and tip radius 20 nm is used. Calibration is performed for each probe using a silicon-carbide calibration specimen with a known coefficient of friction of 0.19. For wet (lubricated) LFM, a tip holder is used to ensure that formed menisci are kept far away from the tip-sample conjunction, thus not affecting the measurements [17].

For each disc sample, its surface is first measured prior to tribometry in nominally dry condition (i.e. virgin surface-dry, relative humidity: $50 \pm 5\%$.), as well as in the presence of a lubricant (i.e. virgin surface-wet). Post tribometry further LFM measurements are carried out in the region of wear tracks, corresponding to different sliding speeds, whilst the disc surface is still wetted (denoted by key identifiers: “value rpm – Wet”). These tests are intended to retain any physisorbed and chemisorbed layers on the surface. The disc is then cleaned with a non-polar solvent (e.g. petroleum ether) to remove any weakly adsorbed physisorbed layer, and the surfaces are left to dry. A final set of LFM measurements are then conducted (denoted by identifiers: “value rpm – Dry”) to determine the nanoscale frictional behaviour of any strongly bonded chemisorbed tribo-film on the wear scar at each of the speed tracks on the surface of disc samples.

**Fig. 6:**
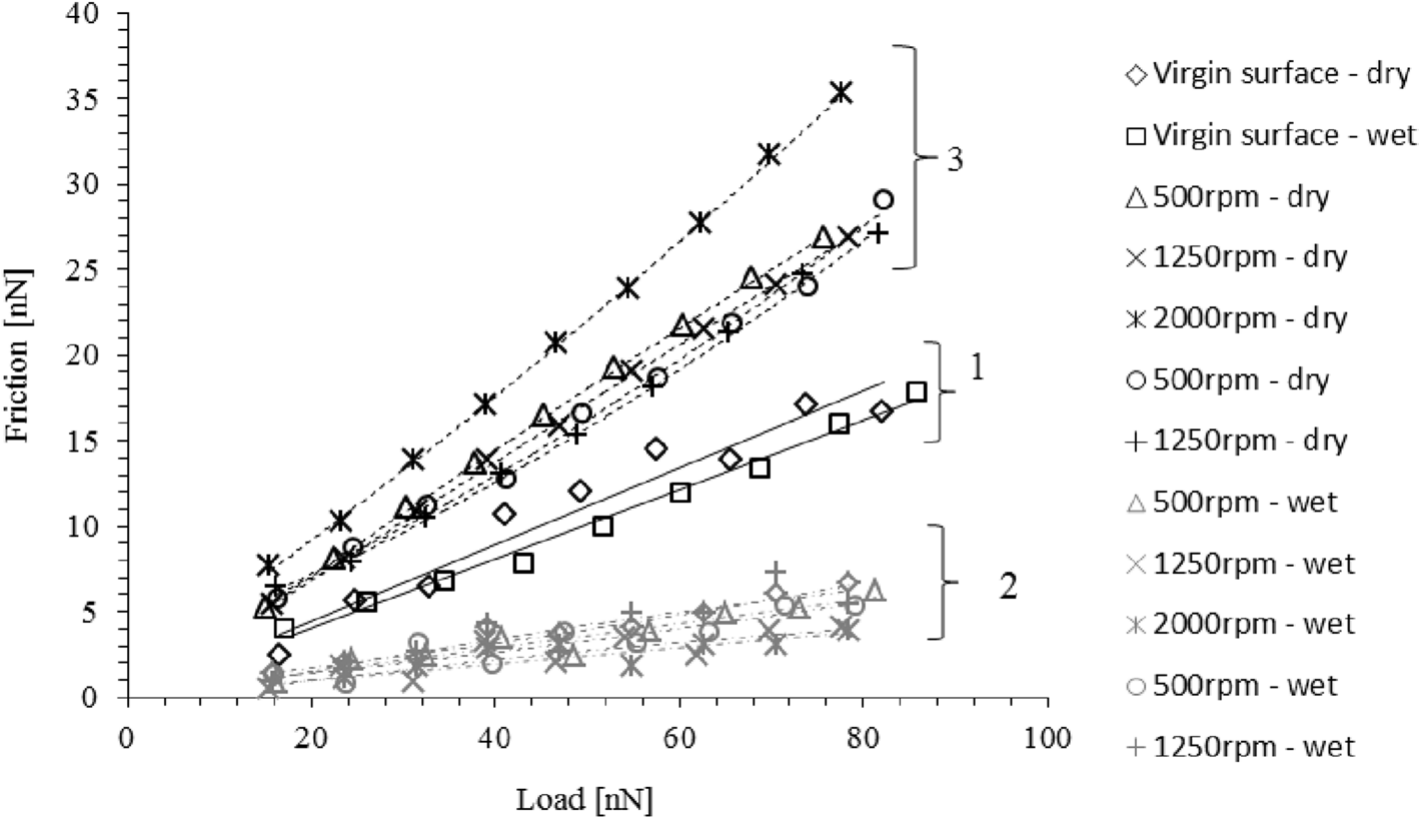
Nanoscale LFM measurements of interfacial boundary shear strength for EN36C disc samples under various conditions

Figure 6 shows the LFM results under various stated conditions. The slope of the lines corresponds to the pressure coefficient of interfacial boundary shear strength of the surface, ${{{\upzeta}}}$. This is in effect the coefficient of friction at asperity interaction level (i.e. mesoscopic or nanoscale).

There are three distinct groups at the different stages of the measurement procedure. These are as follows:

Group 1: shows the results for dry and lubricated contacts of a virgin sample (pre-tribometry) with no thermally or mechanically activated tribo-film. The values of ${{{\upzeta}}}$ are 0.22 (dry) and 0.20 (lubricated). The reduction of friction in the presence of a lubricant is because of reduced shear strength of a fluid film entrained into the contact. The small reduction in the value of ${{{\upzeta}}}$ implies that there is little or no boundary active tribo-film present pre-tribometry.

Group 2: shows the analysis for a lubricated contact (wetted) post tribometry on a disc sample wear tracks. Therefore, the analysis corresponds to the combined effect of any physisorbed and chemisorbed layers of an activated tribo-film. It can be seen that there is a significant reduction in friction, with the value of the pressure coefficient of interfacial boundary shear strength, ${{{\upzeta}}}$ measured in the range of 0.052–0.081, when compared with the lubricated virgin sample (without a formed tribo-film) with a value: $\varsigma =0.203$. This shows that a low friction physisorbed and chemisorbed layer must be formed and retained on the disc wear tracks post tribometry.

Group 3: shows the LFM results for cleaned and air- dried discs after tribometry. The results show increased friction after the removal of the lubricant reside. The dried sample has an increased interfacial shear strength compared with the dried virgin sample (pre-tribometry). This must be due to chemical bonding of surface-active lubricant additives to the surface of disc tracks, subjected to combined shear, pressure, and temperature activation. The measured values of ${{{\upzeta}}}$ are in the range of 0.326–0.447. This finding is in line with those reported in literature for enhanced friction of ZDDP-dominated tribo-films, acting as an extreme pressure additive [13, 14, 26, 28].

**Fig. 7:**
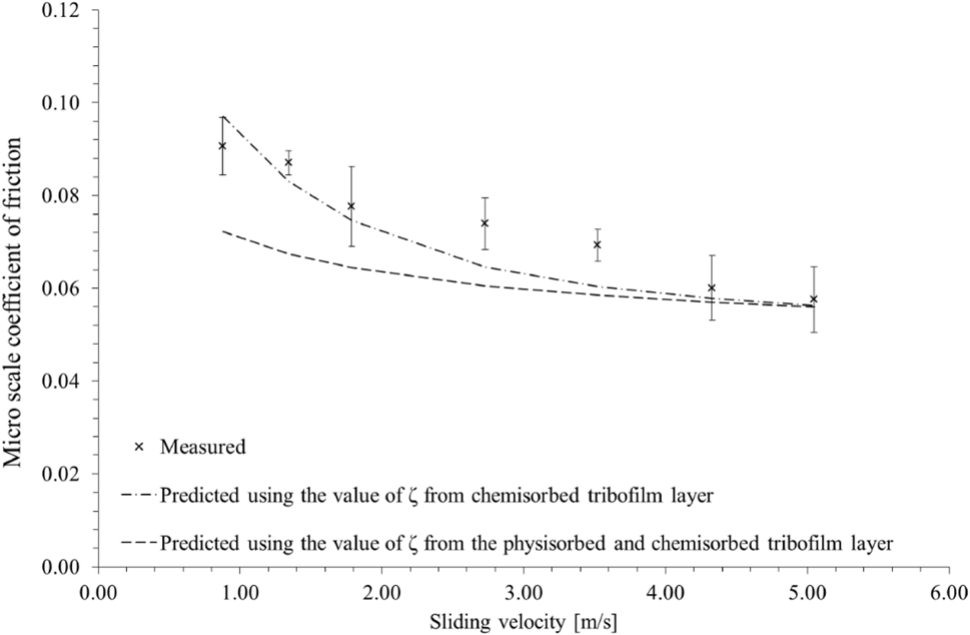
Variation of measured coefficient of friction with sliding velocity and their predictions

Figure 7 shows the variation of measured coefficient of friction from tribometry with varying sliding velocity. The experimental data are compared with the analytical model presented in Sect. 3.2 and 3.3, also using the appropriate pressure coefficient of boundary shear strength, ${{{\upzeta}}}$ measured by LFM (Table 3). At higher speeds, where there is diminutive boundary friction as the result of formation of a coherent elastohydrodynamic lubricant film, the predictions and measurement closely conform. The pressure coefficient of boundary shear strength for the surface with a retained physisorbed and chemisorbed film (group 2) significantly under-predicts the measured outcomes at slower sliding speeds. However, the pressure coefficient of boundary shear strength for the case where only a chemisorbed film is retained (group 3) conforms closely with the measurements.

**Table 3:** Collection of interfacial boundary shear strength coefficients measured by LFM

Condition	Pressure coefficient of boundary shear strength average
0.9 m/s - Dry post tribometry	0.347
2.7 m/s - Dry post tribometry	0.334
5.0 m/s – Dry post tribometry	0.447
0.9 m/s - Lubricated post tribometry	0.070
2.7 m/s - Lubricated post tribometry	0.067
5.0 m/s – Lubricated post tribometry	0.065
Dry virgin surface	0.224
Lubricated virgin surface	0.203

The effect of adsorbed and bonded layers of any formed tribo-film, measured using LFM at nanoscale, can be compared with the micro-scale tribometric measurements. The two measured values for the pressure coefficient of boundary shear strength of a chemisorbed layer provide good correlation with tribometric data, implying that a formed tribo-film and its frictional properties under extreme contact conditions are a function of the regime of lubrication and wear behaviour in micro-scale tribology. This finding also implies that any weakly adsorbed or bonded physisorbed layer in the formed tribo-film is removed through wear, leaving only the chemically absorbed/bonded layer which dominates the boundary frictional characteristics.

### Surface Chemical Analysis

**Fig. 8:**
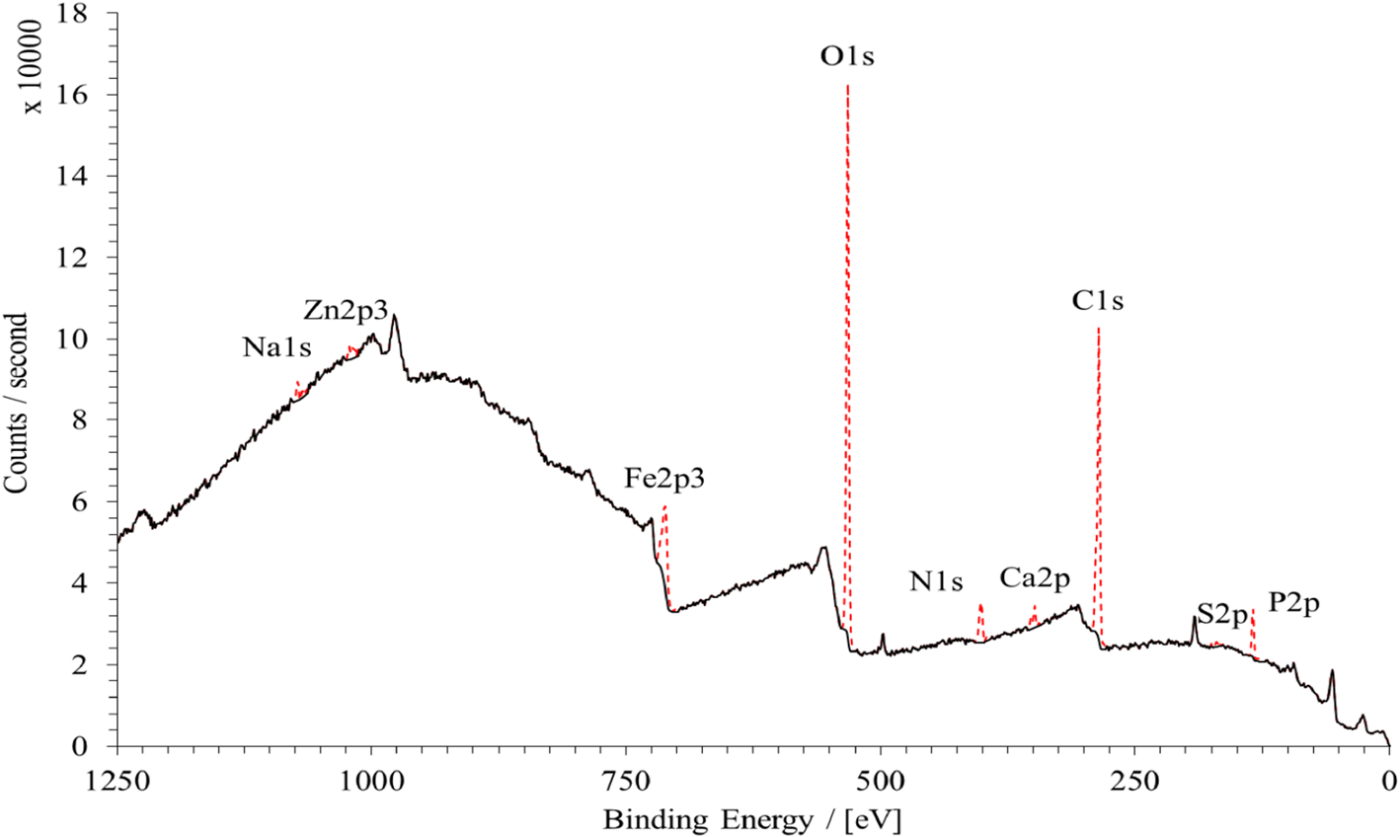
An example spectrum of XPS surface chemical analysis

Using the pin-on-disc tribometer as well as LFM, it is clear that the boundary active lubricant additives are thermo-mechanically activated through the testing procedure. Therefore, analysis of the composition of the bonded/absorbed layers on the surface of disc samples can be carried out using X-ray Photoelectron Spectroscopy (XPS). Disc samples are analysed for an area of 200$\mu {m^2}$ on the wear scar tracks created by the tribometric tests. A survey scan with an extended dwell time is used to determine the chemical composition of the bonded tribo-film. An example of the XPS surface chemical analysis is shown in Fig. 8. The indicated peaks show detection of bonded elements onto the surface of the disc sample wear scar, in addition to the original background spectrum.

**Fig. 9:**
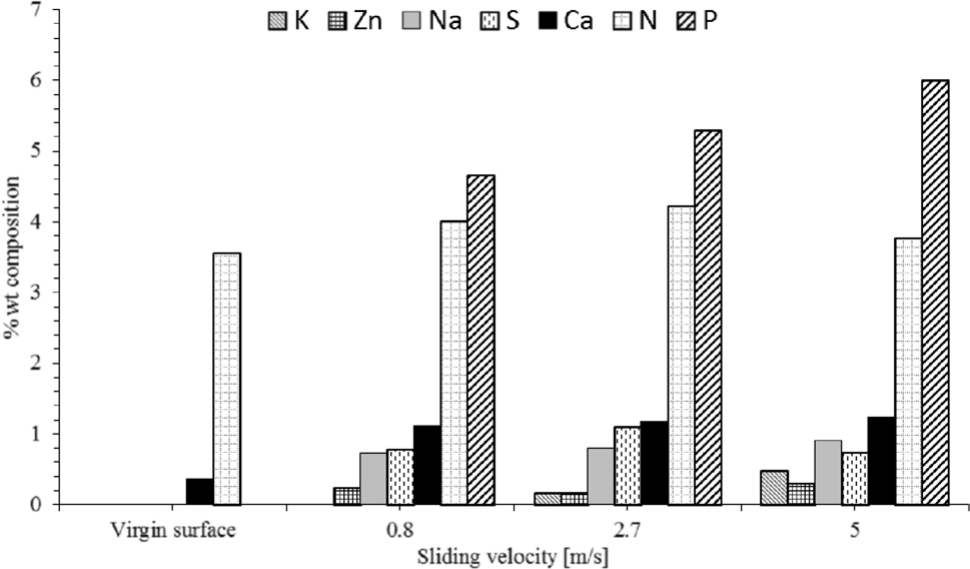
Percentage weight composition of the adsorbed tribo-film post tribometry, note that the rest of the composition is made of carbon and oxygen deposits

There is presence of Potassium, Sodium, Phosphorus, Sulphur and Zinc. All these elements offer performance enhancement as anti-wear, anti-oxidation agents and as friction modifiers [46]. Figure 9 shows a comparison of tribo-film elemental composition for slow, medium and fast sliding velocity test samples. It is clear that different shear rate affects the activation rate of different elements.

**Fig. 10:**
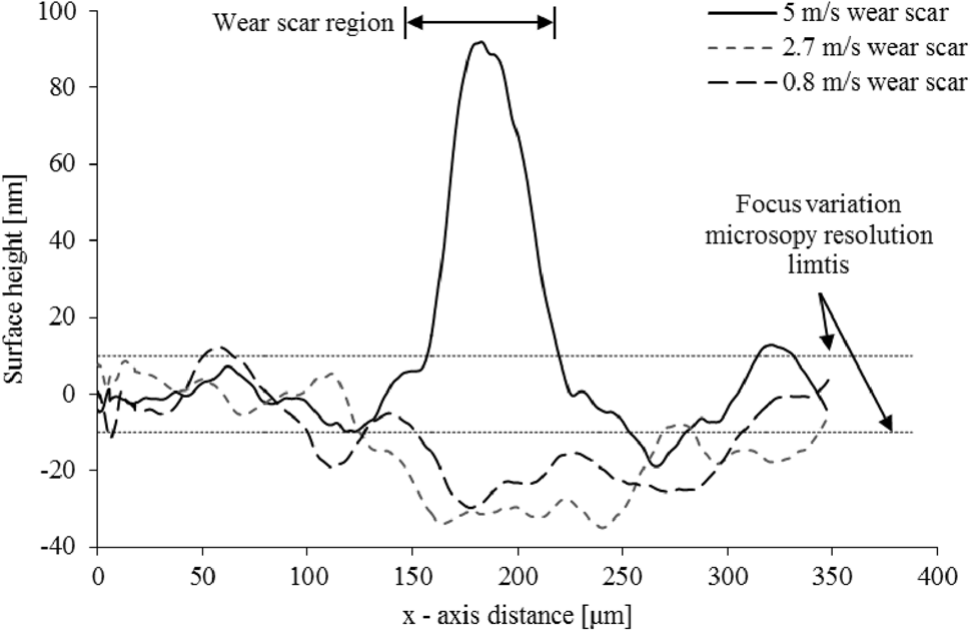
Evolution of surface topography (tribo-film height) post tribometry for varying sliding velocities across the wear track scars

The results in Fig. 9 can be explained through two underlying mechanisms. Firstly, there is an increasing rate of energy generated in the contact with an increasing sliding velocity and available to activate elemental species adsorption/bonding onto the contacting surfaces. There can be an increase in contact oxidation, therefore greater activation of the anti-oxidant additives. Secondly, increased lubricant entrainment speed into the contact enhances the elastohydrodynamic lubricant film thickness, reducing the rate of wear. This is clearly shown in Fig. 10. Tribo-film growth is a complex interaction of film growth and removal through wear [23, 46].

Figure 10 shows the evolution of surface height across the sample disc track wear scars. This is a measure of tribo-film formation through the dual process of growth and wear. It shows that the contact conditions under high sliding velocity allow the deposition of a tribo-film on the surface. In comparison the slower sliding velocities do not show any significant tribo-film growth, but gradual wear of the surface. Therefore, it is clear that with reducing sliding velocity (reduced shear rate), the ensuing boundary regime of lubrication promotes an increased ratio of wear-to-growth of a tribo-film, leading to a gradual removal of the surface. This transition into the boundary regime of lubrication is predicted by the microscale analytical model (Fig. 7). The changing ratio wear-to-growth of a tribo-film means that the physisorbed layer of any formed tribo-film is continually subject to wear. Therefore, the chemisorbed interfacial shear stress determined through use of LFM is more representative for the prevailing contact conditions. Under the slow sliding velocities, the ZDDP acts as an extreme pressure additive as designed; however, the tribo-film is continually removed by the interacting counter face asperities. At high sliding velocities, a full elastohydrodynamic lubricant film is formed with sufficient shear energy to activate the growth of a tribo-film [47]. It should be noted that the surface roughness along the direction of sliding was observed to consistently reduce when compared to the virgin surface, further indicating gradual wear of the surface when no tribo-film is formed and retained.

**Fig. 11:**
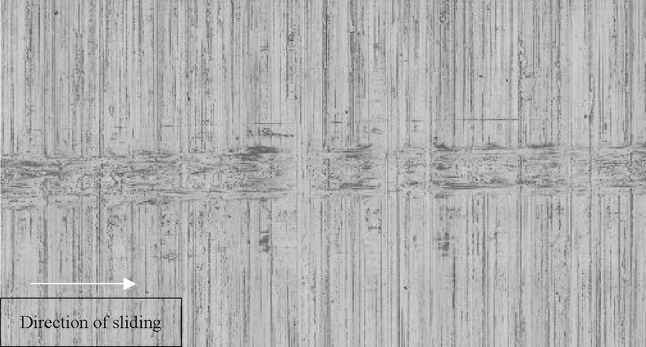
An example of the wear scar for 5 m/s track

The evolution of surface topography is ascertained through a optical focus-variation technique. A x100 magnification optic is used with a measurement area of$~110~ \times 140$ µm^2^. An averaged value is obtained over 10,000 surface profiles perpendicular to the direction of sliding (Fig. 11).

The presence of zinc in XPS results with the evidence of increased boundary shear strength from LFM and growth of wear track height using focus variation microscopy provide strong indication of the presence of the extreme pressure additive ZDDP tribo-film. Furthermore, the discolouration observed in Fig. 11 is documented within literature to be a formed oxide/ sulphide tribo-film and an indication of ZDDP surface additive activation from the lubricant onto the disc surface [48].

## Concluding Remarks

The paper presents a procedure for analysing lubricant-surface combinations for multiscale boundary friction characteristics. It demonstrates the formation of a tribo-film through activation of additives from a transmission system lubricant onto disc samples in pin-on-disc tribometry, subjected to representative material, topographical and operating conditions of high-performance racing transmissions. The boundary frictional properties of the lubricant-surface system are obtained through use of lateral force microscopy, determining the pressure coefficient of interfacial boundary shear strength. The combination of the physisorbed and chemisorbed formed tribo-film layers are critical in the reduction of friction within a lubricant-surface system. Significant differences are observed between the boundary frictional characteristics of chemisorbed and physisorbed layers.

In-depth explanation for experimental measurements is provided through a developed microscale analytical model, linked to the nanoscale interactions through the input of the measured boundary interfacial shear strength of rough surfaces. The results show that the formed tribo-film frictional characteristics are a function of microscale regime of lubrication and wear. This finding is due to the severity of the replicated contact conditions and may be considered to be contact-specific.

## List of Symbols

$A$: Apparent contact area
${A_a}$: Asperity contact area
$a$: Contact footprint semi-half width
$$B$$: Damping coefficient
${c_p}_{{(L)}},~{c_p}_{{(s)}}$: Specific heat capacities of the lubricant and surface material
$$D$$: Deborah number
${E_1},{E_2}$: Elastic moduli of contacting surfaces
${E^{\text{*}}}$: Composite (effective) Young’s modulus of elasticity of the contact
*F*: Applied load
$$f$$: Total friction
$${F_2},{F_{5/2}}$$: Statistical functions
$${f_b}$$: Boundary friction
$${f_v}$$: Viscous friction
$G$: Shear modulus of the lubricant
${G_0}$: Atmospheric shear modulus of the lubricant
${G^{\text{*}}}$: Dimensionless materials’ parameter
${G_e}$: Dimensionless elastic parameter
${G_v}$: Dimensionless viscous parameter
${h_o}$: Central contact lubricant film thickness
${K_{(L)}},{K_{(s)}}$: Thermal conductivities of the Lubricant and sample material
$$p$$: Pressure
${p_0}$: Maximum contact pressure
$\bar {p}$: Average contact pressure
$R$: Effective contact radius (pin contact face radius)
${S_0}$: A constant in lubricant viscosity–temperature relationship
$$U$$: Sliding velocity
${U_e}$: Lubricant entrainment velocity
${U^{\text{*}}}$: Dimensionless speed (rolling viscosity) parameter
$$W$$: Normal load
${W_a}$: Load carried by asperities
${W_v}$: Lubricant viscous reaction
${W^{\text{*}}}$: Dimensionless load parameter
$z$: Pressure-viscosity coefficient

## Greek Symbol

${\alpha _0}$: Piezo-viscosity index
$\beta$: Average asperity tip radius
${\beta _0}$: Thermo-viscosity index
$\eta$: Lubricant dynamic viscosity
${\eta _0}$: Atmospheric lubricant dynamic viscosity
${\mu _v}$: Coefficient of viscous friction
$\mu$: Contact coefficient of friction
${\tau _0}$: Lubricant Eyring shear stress
${\tau _b}$: Shear stress of adsorbed/bonded tribo-film
$\xi$: Average asperity peak height density
${{\upzeta}}$: Pressure coefficient of boundary shear strength
${\Theta}$: Bulk free surface temperature of disc sample
${{{\Theta}}_0}$: Reference temperature at ambient condition
${{{{\Theta}}}_e}$: Effective contact temperature
${{{{\Theta}}}_m}$: Contact temperature rise above that of the bulk
$\sigma$: Composite RMS roughness
$\lambda$: Stribeck’s oil film ratio
${\upsilon _1},{\upsilon _2}$: Poisson’s ratios of contacting surfaces
${\rho _{(s)}}$: Density of disc sample

## Abbreviation

AFM: Atomic force microscope
LFM: Lateral force mode
MTM: Mini-traction machine
SEM: Scanning electron microscope
XPS: X-ray photoelectron spectroscopy
ZDDP: Zinc dialkyldithiophospates
